# 

**Published:** 1846-08

**Authors:** 


### TO READERS AND CORRESPONDENTS.

Communications are on file from Drs. Evans, Ames, Allaire and Huber.

We must ask the indulgence of our Correspondents for any omissions in the acknowledgment of works, &c. received. In consequence of the departure of Dr. Herrick some of them may have been omitted.

We have received also the following publications—

The Medical Examiner. (In Exchange.)

The Bulletin of Medical Science. (In Exchange.)

The Western Journal of Medicine & Surgery. (In Exchange.)

The Buffalo Journal, and Medical Review. (In Exchange.)

Southern Medical & Surgical Journal. (In Exchange.)

The Western Lancet & Medical Library. (In Exchange.)

The St. Louis Medical and Surgical Journal. (In Exchange.)

The Medical News and Library. (In Exchange.)

The American Journal and Library of Dental Science. (In Exchange.)

The Boston Medical and Surgical Journal. (In Exchange.)

The Missouri Medical & Surgical Journal. (In Exchange.)

The New York Medical & Surgical Reporter. (In Exchange.)

The New York Journal of Medicine and the Collateral Sciences. (In Exchange.)

Summary of the Transactions of the College of Physicians.

Annual Announcement of the Medical Department of the University of New York. Session, 1846-47.

Annual Announcement of the Jefferson Medical College, Philadelphia. Session 1846-47.

Catalogue of the Trustees, Faculty and Students of the South Carolina Medical College. Session 1845-46.

Annual Announcement of the Willoughby Medical College. Session 1846-47.

Minutes of the Proceedings of the National Medical Convention, held in the City of New York, May, 1846.

### CONTRIBUTORS TO THE ILLINOIS & INDIANA MED. & SURG. JOUR.

A. H. Howland, M. D , Ottawa, Ill.
A. G. Henry, M. D., Pekin, Ill.
Edward Lewis, M. D., Jackson, Mich.
John McLean, M. D. Jackson, Mich.
Daniel Stahl, M. D., Quincy, Ill.
Edward Mead, M. D. Geneva, Ill.
E. Fisher, Waynesville, Ohio.
H. S. Huber, M. D., Chicago, Ill.
Samuel G. Armor, M. D., Rockford, Ill.
Ira C. Bachus, M. D., Jackson, Mich.
J. G. Cornell, M. D., Spring Arbor, Mich.
G. N. Fitch, M. D., Logansport, Ind.
John F. Henry, M. D., Burlington, Iowa.
E. Deming, M. D., Lafayette, Ind.
S. B. Thayer, Battle Creek, Mich.

### ADVERTISEMENT.

### UNIVERSITY OF THE STATE OF NEW YORK.

### COLLEGE OF PHYSICIANS AND SURGEONS.

### FORTIETH SESSION,

THE Annual Course of Lectures, in the College, will be commenced on Monday November 2, 1846, and continued until March 1, 1847.

ALEXANDER H. STEVENS, M. D., President of the College, and Emeritus Professor of Clinical Surgery.

JOSEPH MATHER SMITH, M. D., Professor of the Theory and Practice of Medicine, and Clinical Medicine.

JOHN B. BEACH, M. D., Professor of Materia Medica, and Medical Jurisprudence.

JOHN TORREY, M. D., L. L. D., Professor of Chemistry, and Botany.

ROBERT WATTS, jr., M. D., Professor of Anatomy and Physiology.

WILLARD PARKER, M. D., Professor of the Principles and Practice of Surgery and Surgical Anatomy.

CHANDLER R. GILMAN, M. D., Professor of Obstetrics and the Diseases of Women and Children.

GUSTAVUS A. SABINE, M. D., Demonstrator of Anatomy.

*Fees.* Matriculation Fee, $5. Fees for the full Course of Lectures, $94. Demonstrator's Ticket, $5. Graduation Fee, $25 Board, average per week, $3. Clinical Instruction is given at the New York Hospital daily, by the Medical Officers, (Professor Smith being one of them,) fee, $8, per annum: at the Eye Infirmary, without fee; and about 1000 patients are annually exhibited to the Class in the College. Clinical, Obstetrical cases and Anatomical *materiel* are abundantly furnished through the respective departments.

The annual commencement is held on the second Thursday in March; there is also a semi annual examination in September. The requisites for graduation are, twenty-one years of age, three years of study, including two full courses of lectures, the *last* of which, must have been attended in this College, and the presentation of a Thesis on some subject connected with medical science.

During the month of October, a course of lectures will be delivered on the following subjects;

| | |
|---|---|
| Hygiene, - - - - - | by Professor SMITH. |
| Comparative Anatomy, - - - - | " Professor WATTS. |
| Venereal Diseases, - - - - | " Professor PARKER. |
| Diseases of the Os and Curvix Uteri, - | " Professor GILMAN. |

This course will be *free* to the *Matriculated* students of the College.

College of Physicians and Surgeons, No. 67, Crosby street, New York.

R. WATTS, jr., M. D., Secretary to the Faculty.

